# Distinguishing Acute Pancreatitis From Refractory Peritonitis in Peritoneal Dialysis: A Case Report

**DOI:** 10.7759/cureus.95093

**Published:** 2025-10-21

**Authors:** Joaquim Milheiro, Raquel Pinto, Cátia Pêgo, Tania Sousa, Sérgio Lemos

**Affiliations:** 1 Nephrology Department, Unidade Local de Saúde (ULS) Viseu Dão-Lafões, Viseu, PRT

**Keywords:** acute pancreatitis (ap), diagnostic approach, end-stage kidney disease (eskd), peritoneal dialysis complications, peritoneal dialysis related peritonitis

## Abstract

Patients undergoing peritoneal dialysis (PD) are at increased risk for acute pancreatitis, a rare but potentially fatal disease that arises from still poorly understood mechanisms. We report the case of a 60-year-old woman on PD admitted to the emergency room with severe epigastric pain, nausea, and emesis. The patient, suffering from stage 5 chronic kidney disease (CKD) secondary to autosomal dominant polycystic kidney disease, was undergoing treatment for PD-related peritonitis. She presented on day 17 of peritonitis treatment with a cloudy peritoneal effluent and elevated pancreatic enzymes. Exclusion of classical etiologies for pancreatitis was key in ensuring an accurate diagnosis. A thorough anamnesis may identify possible triggers or risk factors involved in the development of this pathology. Peritoneal effluent evaluation is one of the most trustworthy monitoring tools. Knowledge of similar cases is warranted to further understand how we can foresee or even prevent some of these episodes, as well as their complications.

## Introduction

Peritoneal dialysis (PD) is a widely used kidney replacement therapy for patients with end-stage kidney disease. Despite its benefits, PD patients are susceptible to several complications, including infections, gastrointestinal disorders, and, less commonly, acute pancreatitis [1, 2]. The underlying mechanism for this propensity has yet to be explained. Several hypotheses have been proposed, including direct effects of dialysis solutions on pancreatic enzyme activation, uremic toxin accumulation, altered calcium homeostasis, and secondary hyperparathyroidism [2, 3]. Additionally, the immunocompromised state of dialysis patients may predispose them to infectious complications that could contribute to pancreatic inflammation. Cloudy or hematic peritoneal effluent can be particularly concerning, warranting a thorough and timely differential diagnosis to identify the underlying cause [2, 4]. These can include PD-associated peritonitis (PD peritonitis), hemoperitoneum, pancreatitis, PD catheter complications, intra-abdominal malignancies, and ruptured ovarian cysts. Coexisting inflammatory processes can make peritoneal effluent characteristic interpretation challenging.

The incidence of acute pancreatitis in dialysis patients varies considerably across studies and geographic regions. Large epidemiological studies have reported incidence rates ranging from 18.4 to 38.2 per 100,000 patients in American and European populations [5-7]. A comprehensive 10-year Taiwanese cohort study found an incidence of 5.8 per 1000 person-years in end-stage renal disease patients, representing a substantially increased risk compared to the general population [8]. Notably, these studies consistently demonstrate higher incidence rates in PD patients compared to hemodialysis patients, suggesting unique risk factors associated with PD therapy.

Diagnosis of acute pancreatitis in PD patients presents unique challenges, particularly when clinical presentation overlaps with other common complications such as peritonitis. The presence of cloudy or blood-tinged peritoneal effluent may occur in both conditions, necessitating careful differential diagnosis. Furthermore, the interpretation of pancreatic enzymes may be complicated by altered clearance in renal failure and potential interference from certain dialysis solutions.

## Case presentation

A 60-year-old woman with stage 5 chronic kidney disease (CKD) secondary to autosomal dominant polycystic kidney disease presented to the emergency department in November 2023 with severe epigastric pain, nausea, and vomiting. She had been in automated PD (APD) for one year, using glucose-based solutions (four cycles of 1.5% glucose, 1500 ml each) with adequate dialysis efficacy. Her CKD complications, including anemia and secondary hyperparathyroidism, were well-controlled with appropriate medical therapy. Her pharmacological prescription included furosemide 80 mg tid, amlodipine 5 mg id, omeprazole 20 mg id, alfacalcidol 0.5 mcg id, iron dextran 100 mg weekly, and erythropoietin 2000 IU weekly.

At presentation, the patient was on day 17 of antibiogram-guided treatment with intraperitoneal ceftazidime for *Enterobacter ludwigii *peritonitis. Her peritonitis had shown a good clinical response, with the peritoneal effluent leukocyte count decreasing from 1258/μl to 91/μl over five days of treatment.

The patient had experienced two additional infectious episodes in the preceding three months: a bacteremic urinary tract infection caused by *Escherichia coli* with associated peritonitis, treated for three weeks with intraperitoneal ceftazidime, and a *Clostridioides difficile* colitis treated with oral vancomycin and metronidazole for 10 days. During both infectious episodes, she had been managed through a home hospitalization program with a temporary transition from APD to continuous ambulatory PD (CAPD) with icodextrin night dwells to facilitate treatment delivery.

On admission, vital signs were stable: blood pressure 130/80 mmHg, heart rate 76 beats per minute (bpm), respiratory rate 18 breaths/min, and temperature 36.8°C. Physical examination revealed abdominal distention with severe epigastric tenderness on palpation, without muscular guarding. Notably, the peritoneal effluent was blood-tinged and cloudy, raising immediate concern for complications of either peritonitis or an alternative abdominal process.

Initial laboratory investigation, as shown in Table 1, found normal blood cell count, C-reactive protein (CRP), and procalcitonin. Lipid and liver profiles were normal. Both serum amylase and lipase were greater than threefold the upper reference limit. The peritoneal effluent leukocyte cell count showed 213/μl with a predominance (60%) of monocytes.

**Table 1:** Initial laboratory findings PTHi: intact parathyroid hormone; CRP: C-reactive protein; K: potassium; Na: sodium; Ca: calcium; P: phosphorus; ALT: alanine aminotransferase; AST: aspartate aminotransferase; ALP: alkaline phosphatase; GGT: gamma-glutamyl transferase; LDH: lactate dehydrogenase; PE: peritoneal effluent

Parameter	On admission	Lab reference value	Units
Hemoglobin	11.3	12-15	g/dl
White blood cell count	7.2	4.5-11.5	x10^3^/μl
Platelet count	240	150-450	x10^3^/μl
PTHi	181	105-580	pg/ml
Serum amylase	213	13-53	U/l
Serum lipase	1467	6-51	U/l
Triglyceridemia	72	< 150	mg/dl
Total bilirubin	0.3	0.2-1.1	mg/dl
Glucose	125	74-106	mg/dl
CRP	5	< 0.5	mg/dl
Procalcitonin	0.1	< 0.5	ng/ml
K	4.5	3.5-5.5	mEq/l
Na	135	135-145	mEq/l
Ca (48 hours post admission)	9	8.2-10.3	mg/dl
P	3.8	3.5-5.5	mg/dl
ALT	20	3-31	IU/l
AST	18	3-31	IU/l
ALP	82	25-100	IU/l
GGT	25	< 38	IU/l
LDH	234	120-245	IU/l
PE leukocyte count	213	-	/μl
PE erythrocyte count	202	-	/μl

The patient's electrolytes, kidney function tests, and other routine blood investigations were within expected and adequate ranges for a patient on dialysis. Her last small solute dialysis clearance had a standardized kt/v of 1.9.

An abdominal ultrasound was performed, which showed no evidence of gallstones, bile duct dilation, or other obstructive causes. The pancreatic structure was poorly visible but appeared normal without signs of acute inflammation or fluid collections. Given the patient’s clinical stability and ultrasound findings, additional imaging was deferred.

Clinical stability at 48 hours with ease in pain and no further elevation in peritoneal effluent cell count discouraged the performance of any computerized tomography scan. The performance of such an exam was again pondered at day 7 of inpatient care but deemed unnecessary in light of symptom resolution and a resumed tolerated diet. No other imaging exams were performed.

She was admitted to the hepatobiliary unit with the diagnosis of acute pancreatitis with a differential diagnosis of refractory infectious peritonitis, inflammatory pancreatitis-related peritonitis, and drug-related peritonitis. The patient was admitted to the surgical unit for conservative management, including bowel rest, intravenous fluids, analgesics, and close monitoring. She completed her prescribed course of intraperitoneal ceftazidime while maintaining PD therapy. Regular monitoring of peritoneal effluent cytology showed progressive improvement.

**Figure 1:**
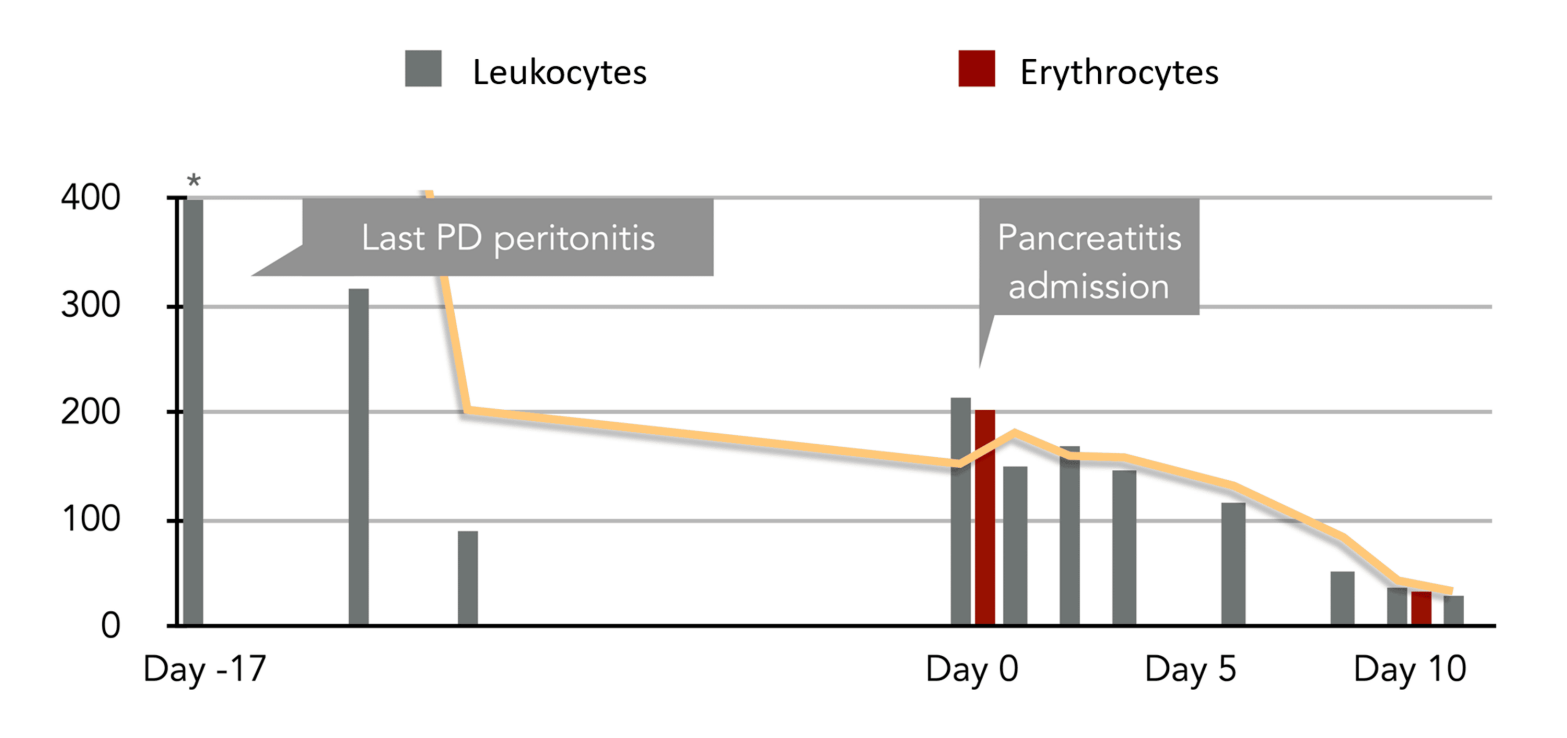
The evolution of peritoneal effluent cell count (/μl) PD: peritoneal dialysis

By day 9 of hospitalization, complete clinical recovery with normalization of pancreatic enzymes (lipase and amylase returned to normal limits), reduction of peritoneal effluent leukocyte count to 39/μl (full peritoneal effluent cell count shown in Figure 1), and resolution of abdominal pain were achieved. No complications occurred during the treatment course.

The dialysis prescription was kept unchanged with a daily ultrafiltration ranging from 1400 to 2000 ml. She was discharged on day 10 with no pain, normalized amylase and lipase, and resumed her PD at home.

## Discussion

Diagnosing acute pancreatitis occurring during active treatment for PD peritonitis is complex, taking into account the overlap of the main signs and symptoms. According to the revised Atlanta classification, acute pancreatitis diagnosis requires two of three criteria: characteristic abdominal pain, serum lipase/amylase ≥3 times the upper normal limit, and characteristic imaging findings [9, 10]. Our patient clearly met the first two criteria, with imaging studies excluding alternative diagnoses.

The presence of blood-tinged peritoneal effluent initially raised concern for hemorrhagic pancreatitis, which could indicate severe disease. However, the patient’s stable clinical condition, normal inflammatory markers, and benign imaging findings suggested mild pancreatitis without complications.

Blood-tinged peritoneal effluent in PD patients requires systematic evaluation to exclude serious complications. In this case, the differential diagnosis included hemorrhagic pancreatitis with peritoneal inflammation, infectious peritonitis with vascular permeability changes, catheter-related trauma or malposition, intra-abdominal malignancy or ruptured visceral structures, and anticoagulation-related bleeding.

Infectious, pharmacological, and PD-related factors may have contributed to pancreatitis development in this patient. The patient’s recent history of multiple abdominal infections, including *Enterobacter ludwigii* peritonitis, could have triggered pancreatic inflammation through direct infection or systemic inflammatory responses [11]. While uncommon, bacterial translocation or direct pancreatic involvement cannot be entirely excluded in immunocompromised dialysis patients. The patient’s exposure to multiple antibiotics, including metronidazole and ceftazidime, raises the possibility of drug-induced pancreatitis. Although beta-lactam antibiotics are rarely associated with pancreatitis, metronidazole has been reported as a potential causative agent in several case reports [12-15]. Chronic exposure to PD solutions may contribute to pancreatitis through various mechanisms, including calcium exposure, solution acidity, osmolarity effects, and chemical irritation leading to premature pancreatic enzyme activation. Additionally, uremic toxins and secondary hyperparathyroidism, though well-controlled in this patient, represent theoretical risk factors.

The concurrent elevation of pancreatic enzymes, clinical presentation, and negative peritoneal fluid cultures supported acute pancreatitis as the primary diagnosis.

Management of acute pancreatitis in PD patients requires modifications to address the unique challenges in this population. Key considerations include fluid management with careful fluid balance for hemodynamic stability while avoiding volume overload and continued PD for adequate fluid removal and facilitated completion of antibiotic therapy. Maintaining CAPD during the acute episode proved beneficial for both infection treatment and potential removal of inflammatory mediators from the peritoneal cavity. Concurrent antibiotic therapy was continued based on the ongoing peritonitis treatment, with close monitoring for clinical response and potential drug-related adverse effects.

PD patients with acute pancreatitis face increased mortality risk compared to the general population. However, early recognition and appropriate management can lead to excellent outcomes, as demonstrated in this case. The patient’s complete recovery within nine days without complications suggests that mild, non-necrotizing pancreatitis in PD patients can have a favorable prognosis with conservative management.

## Conclusions

Acute alithiasic pancreatitis represents a rare but important complication in peritoneal dialysis patients. This case demonstrates that pancreatitis can occur during active treatment for other abdominal complications, potentially complicating diagnosis and management. The presence of blood-tinged peritoneal effluent should prompt careful evaluation for pancreatitis, particularly when accompanied by appropriate clinical symptoms and biochemical abnormalities.

Maintaining a strong clinical suspicion for pancreatitis is important in PD patients who have persistent abdominal pain, even when receiving treatment for other pathologies. When blood-tinged peritoneal effluent is present, a systematic evaluation is vital to rule out serious complications. A broad and nuanced approach to etiology, including both infectious and pharmacologic factors, is essential. Timely recognition and appropriate conservative measures can lead to positive outcomes.

Further research is needed to better understand the pathophysiology of pancreatitis in PD patients and to develop evidence-based management guidelines for this vulnerable population. A multidisciplinary approach involving nephrologists, surgeons, and critical care doctors remains essential for optimal patient outcomes.
